# Real-Time *In Vivo* Detection and Monitoring of Bacterial Infection Based on NIR-II Imaging

**DOI:** 10.3389/fchem.2021.689017

**Published:** 2021-06-14

**Authors:** Sijia Feng, Huizhu Li, Chang Liu, Mo Chen, Huaixuan Sheng, Mingru Huang, Yunxia Li, Jun Chen, Jian Zhang, Yuefeng Hao, Shiyi Chen

**Affiliations:** ^1^ Sports Medicine Institute of Fudan University, Department of Sports Medicine, Huashan Hospital, Fudan University, Shanghai, China; ^2^ Department of orthopedics, Affiliated Suzhou Hospital of Nanjing Medical University, Suzhou, China

**Keywords:** bioimaging, *in vivo* imaging, NIR-II, bacterial infection, quantum dots

## Abstract

Treatment according to the dynamic changes of bacterial load *in vivo* is critical for preventing progression of bacterial infections. Here, we present a lead sulfide quantum dots (PbS QDs) based second near-infrared (NIR-II) fluorescence imaging strategy for bacteria detection and real-time *in vivo* monitoring. Four strains of bacteria were labeled with synthesized PbS QDs which showed high bacteria labeling efficiency *in vitro*. Then bacteria at different concentrations were injected subcutaneously on the back of male nude mice for *in vivo* imaging. A series of NIR-II images taken at a predetermined time manner demonstrated changing patterns of photoluminescence (PL) intensity of infected sites, dynamically imaging a changing bacterial load in real-time. A detection limit around 10^2^–10^4^ CFU/ml was also achieved *in vivo*. Furthermore, analysis of pathology of infected sites were performed, which showed high biocompatibility of PbS QDs. Therefore, under the guidance of our developed NIR-II imaging system, real-time detection and spatiotemporal monitoring of bacterial infection *in vivo* can be achieved, thus facilitating anti-infection treatment under the guidance of the dynamic imaging of bacterial load in future.

## Introduction

Despite the rapid development of antibiotics, bacterial infection remains a major threat to human health, causing massive mortality and morbidity. Due to frequent failures to identify bacterial infections at an early stage *in vivo* with high sensitivity and accuracy, the infection can progress rapidly into a systemic disease along with irreversible damages, even leading to life-threatening conditions due to delayed treatment (Moore, 1993; Erasmus et al., 1999; Ning et al., 2011). Traditionally, diagnosis of bacterial infections mainly relies on *in vitro* approaches such as specimen culture, polymerase chain reaction (PCR), and enzyme linked immunosorbent assay (ELISA), which are not only time-consuming, but also lacking in sensitivity, not to say able to image the infection accurately *in vivo* (Gracias and McKillip, 2004; Heo and Hua, 2009; Fournier et al., 2013; Ahmed et al., 2014). Therefore, an imaging technology enabling early detection of bacterial infections *in vivo* is in urgent need.

For detecting bacterial infections *in vivo*, an ideal approach should firstly be non-invasive and biocompatible with the human body. Besides, an excellent signal-to-noise ratio (SNR) should be guaranteed to avoid interference from the complex dynamic environment inside the body. Furthermore, the overall imaging process should be cost-effective enough for general clinical applications in the future. Recently, fluorescence imaging based on the second near-infrared (NIR-II) window (1,000–1,700 nm) is gaining popularity for *in vivo* imaging due to minimized scattering and high SNR (Liu et al., 2009a; Liu et al., 2009b; Smith et al., 2009; Welsher et al., 2011). As a representative, lead sulfide quantum dots (PbS QDs) have been reported to be a promising semiconductor nanocrystal with excellent NIR-II fluorescence characteristics (Bruchez et al., 1998; Chan and Nie, 1998; Medintz et al., 2003; Wu et al., 2003; Michalet et al., 2005; Chen et al., 2016a), showing a huge potential for *in vivo* imaging with deep tissue penetration and low cytotoxicity (Chen et al., 2016a; Feng et al., 2016).

**SCHEME 1:**
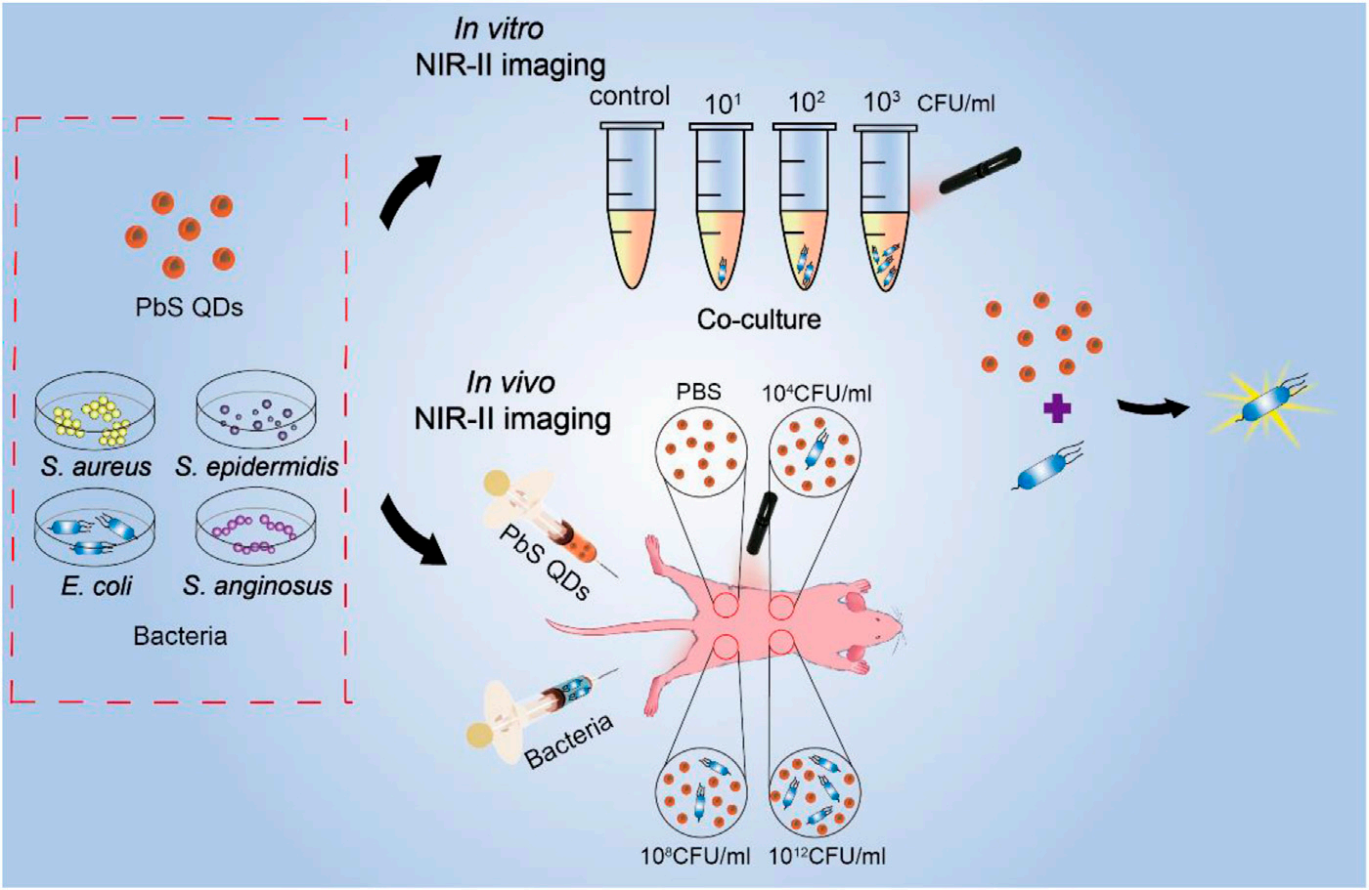
Schematic to represent the experimental process.

Here, we present a NIR-II imaging strategy based on fluorescence PbS QDs, facilitating dynamic detection of bacterial infection both *in vitro* and *in vivo* in a real-time manner (Scheme 1). Firstly, fluorescence properties of PbS QDs were validated. Then, the PbS QDs were co-cultured with three concentrations of four different bacteria strains including *Staphylococcus aureus* (*S. aureus*), S*taphylococcus epidermidis* (*S. epidermidis*), *Escherichia coli* (*E. coli*) and *Streptococcus anginosus* (*S. anginosus*) *in vitro* and highly efficient bacterial labeling was achieved. During *in vivo* observation, the PbS QDs displayed a quick detection of bacteria at the infected sites and allowed a real-time investigation on the change of bacterial load in a longitudinal manner. Besides, the biocompatible PbS QDs were eventually metabolized and cleared out of the body with no noticeable abnormality in major organs. Our work demonstrated a highly efficient probe for bacterial detection and a possible strategy to realize real-time observation and monitoring of bacterial infection *in vivo*, which provides novel thoughts into antibiotic treatment according to bacterial load.

## Materials and Methods

### Reagents and Materials

All chemicals were used without further modification or purification procedure. Deionized water was utilized to arrange the working solutions. Lead acetate trihydrate [Pb(OAc)_2_·3H_2_O, ≥99.9%], sodium sulfide nonahydrate (Na_2_S·9H_2_O, ≥ 98.0%), bovine pancreatic ribonuclease A (MW:13.7 kDa, > 70 U/mg) and sodium hydroxide (NaOH, ≥ 98.0%) were purchased from Sigma-Aldrich. Aseptic phosphate buffer solutions (1 × PBS, pH = 7.4) was used in all experiments.

### Bacteria

The bacteria were provided by the Institute of Antibiotics, Huashan Hospital, Fudan University, Shanghai, including *S. aureus* (ATCC 29213), *E. coli* (ATCC 25922), *S. epidermidis* (clinical isolates), and *S. anginosus* (clinical isolates).

### Animals

Eight-week-old male nude mice with the weight of 20–24 g were purchased from Shanghai Jie Si Jie Laboratory Animal Co. Ltd. (Shanghai, China). All animal studies were done complying with guidelines of the Chinese Council for Animal Care.

### Instrumentation

ELGA Purelab classic UVF system was used to generate deionized water. Microwave synthesis process was performed in a microwave reactor (Discover, CEM). The PbS QDs were neutralized and purified with Amicon ultracentrifugal filter tubes (MWCO: 10 kDa). The centrifugation was done with Thermo Scientific Heraeus Fresco 21 microcentrifuge and/or an Eppendorf 5810R centrifuge. NIR-II fluorescence spectra measurement was carried out with NS1 NanoSpectralyzer fluorimetric analyzer (Applied NanoFluorescence) and the fluorescence excitation wavelength is 785 nm. All NIR-II images of test tubes and mice were taken with a NIR-II imaging prototype connected with a 2D InGaAs CCD camera. 810 nm diode laser coupled with 850 nm and 1,000 nm short-pass filters was utilized as the excitation source. The emission light was filtered with an 1,100 nm long-pass filter before it was detected by the CCD sensor.

### Synthesis of RNase-A@PbS QDs

500 μl of RNase-A (50 mg/ml) solution and 500 μl of Pb(OAc)_2_ (10 mM) solution was mixed to prepare RNase-A/Pb^2+^ precursor solution. 50 μl of NaOH solution (1 M) was added to adjust the pH to 9–11. Then 50 μl of freshly prepared Na_2_S (10 mM) solution was added to the mixture after the solution was stirred and mixed for 5 min. Subsequently a stir bar was put into the mixed solution and the test tube was inserted into the microwave reactor and heated at 70°C for 30 s with an input power of 30 W. The transparent dark brown mixtures can be seen when RNase-A@PbS QDs were formed. Then the PbS QDs were ultra-filtered against deionized water in order to adjust system pH to 7–8 and remove extra reagents (e.g. Pb^2+^, Na^+^, OAc^−^, etc.). After that, PBS was used to resuspend the solution to original volume. The final solution was stored at 4°C and kept away from light.

### Bacteria culture

Four strains of bacteria including *S. aureus*, *E. coli*, *S. epidermidis*, and *S. anginosus* was used to investigate the labeling function of PbS QDs. Bacteria were cultured overnight in Luria-Bertani (LB) medium at 37°C under 5% CO_2_ in an incubator shaker with a set of 150 rpm. Then a single colony was collected and inoculated into a 6 ml of fresh LB medium and cultured to an optical density of 0.5–0.6 at 600 nm in a 10 ml flask. Suspensions of the bacteria were centrifuged at 2000 rpm for 15 min at 25°C. Then the bacterial sediments were collected after being washed by PBS twice and further resuspended in 5 ml of PBS. Finally, the suspensions of each strain of bacteria were diluted respectively into different concentrations for being inoculated to different culture media to calculate the colony-forming units (CFU) per milliliter.

### Co-Culture of Bacteria and PbS QDs

A 50 μl portion of each bacteria strain at concentrations of 10^3^, 10^2^, 10 CFU/ml was added to a 1.5 mlcentrifuge tube and stirred with 50 μl of previously prepared PbS QDs respectively. Then the mixtures of PbS QDs and bacteria were incubated at 37°C under 5% CO_2_ for 1 h for co-culture. After that, the mixtures were centrifuged at 2,000 rpm for 10 min at 25°C. The transparent supernatants were separated from the sediments and removed to new 1.5 ml centrifuge tubes while the sediments were washed by PBS twice. Finally, NIR-II images were taken of the bacterial sediments in comparison with the original supernatants.

### Preparation of Experimental Animal Models

Male nude mice which have body weight ranging from 20 to 24 g were included in all animal experiments. Four nude mice were randomly chosen to be injected with the four strains of bacteria. The nude mice were deeply anesthetized by intraperitoneal injection, then each of them was subcutaneously injected with 25 μl of one strain of bacteria on the back at three sites away from another at a bacterial concentration of 10^12^ CFU/ml, 10^8^ CFU/ml and 10^4^ CFU/ml. The injected sites were at the same positions across the four nude mice. Moreover, a fourth site was injected with the same amount of PBS as a control for each nude mouse.

### *In Vivo* Observation

Right after the four nude mice were injected with bacteria, 50 μl of PbS QDs were injected subcutaneously at the same four sites on the back. Then the four nude mice were kept in cage under constant observation and the same circumstances. NIR-II images were collected from each nude mouse at 10 min, 1 h, 2 h, 1 day, 2 days, 1 w, 2 w and 3 w after the injection. The PL intensity and area of fluorescence of four injected sites of each nude mouse were measured by analyzing every NIR-II image using ImageJ. The data of 2 days post-infection were chosen to be further analyzed for detection limit of bacteria load *in vivo* with regression graphs applied to study the relevance between bacteria load and PL intensity using Graphpad Prism 9. To eliminate the potential background interference at each time point, PL intensity at the injected site/PL intensity of the background was calculated, marked as relative PL intensity.

### Histological Analysis With Hematoxylin-Eosin Staining and Gram Staining

After 3 weeks, skin tissues of the sites with 10^12^ CFU/ml bacteria and the sites with PBS and major organs of the nude mice were collected and stored in 4% paraformaldehyde for at least 24 h to prepare pathological slides for histological analysis. Additionally, NIR-II images of collected major organs were taken and PL intensity was measured before preparing for histological analysis. After H&E and Gram staining were done, histological images were acquired on an optical microscope (Nikon Eclipse CI, Japan). Then immunohistochemical analysis including neutrophil count was done with immunohistochemical slides by calculating average number of neutrophils per square millimeter under three fields of 200× magnification using Image-pro plus 6.0 (Media Cybernetics, Inc., Rockville, MD, United States). Inflammatory factors including CD11b, IL-6, TNF-α, and monocyte chemoattractant protein-1 (MCP-1), which are highly associated with bacterial infection, were analyzed with immunohistochemical technique. Average Optical (AO) was calculated (AO = IOD/AREA, IOD: Integrate Optical Density).

## Results and Discussion

### Fluorescence Properties of PbS QDs

**FIGURE 1:**
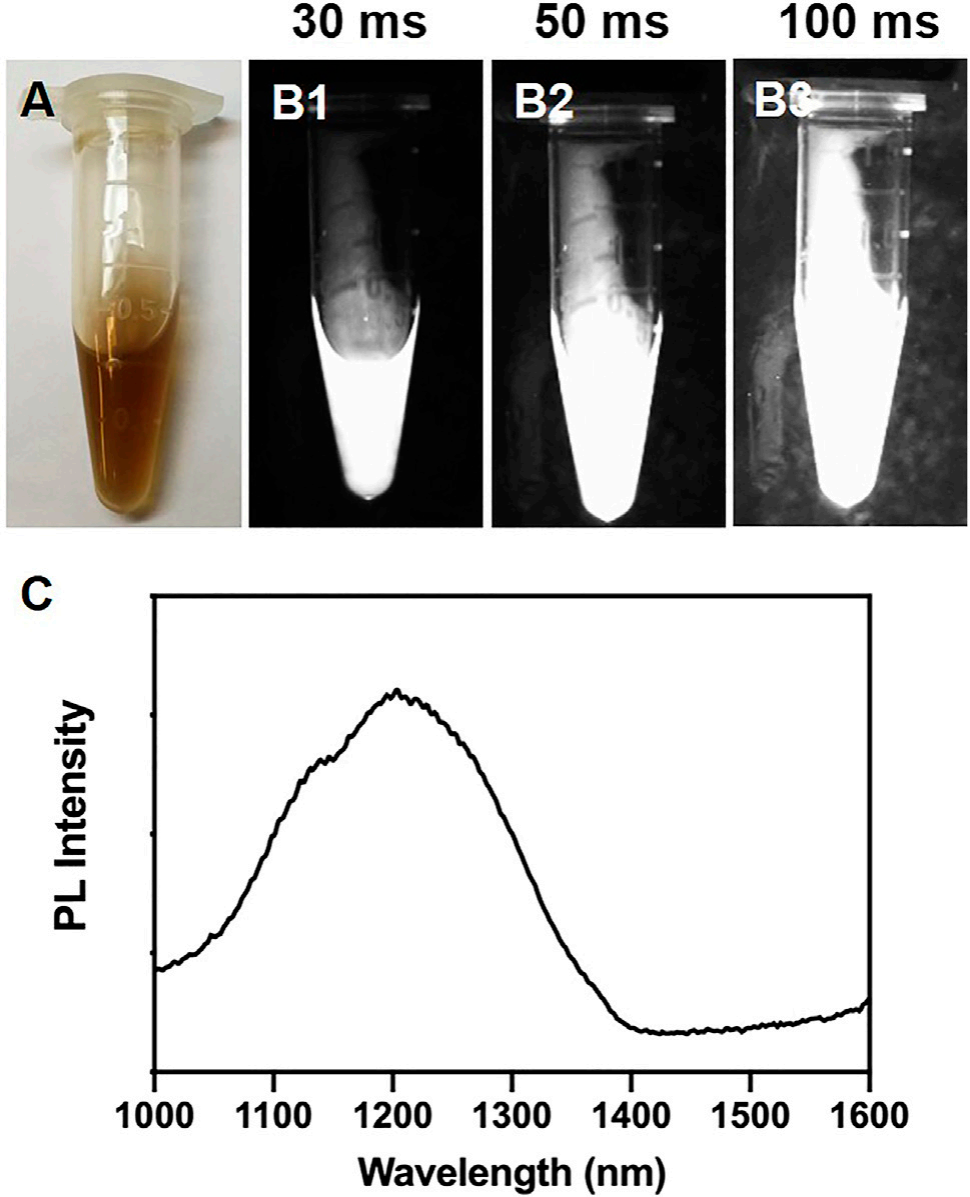
**(A)** Bright-field photograph of the prepared PbS QDs. **(B1–B3)** NIR-II fluorescence images of **(A)** under an exposure time of 30 ms, 50 ms and 100 ms. **(C)** PL intensity of the prepared PbS QDs.

Firstly, PbS QDs were prepared according to the previous protocol (Kong et al., 2016). As shown in Figure 1A, a uniform light-brown liquid without sediments was formed. Then, NIR-II images of the freshly-prepared PbS QDs were achieved with a specialized system (Figures 1B1–B3). Under an increasing exposure time from 30 ms to 100 m, increasing fluorescence signals of the PbS QDs were detected, while the SNR kept low, which was in accordance with previous studies (Welsher et al., 2011; Li et al., 2014; Hong et al., 2015; Chen et al., 2016b; Wan et al., 2018). Furthermore, the photoluminescence (PL) spectrum of the PbS QDs was measured in Figure 1C, revealing an emission peak within the range of NIR-II window.

### *In Vitro* Labeling Efficiency of PbS QDs

**FIGURE 2:**
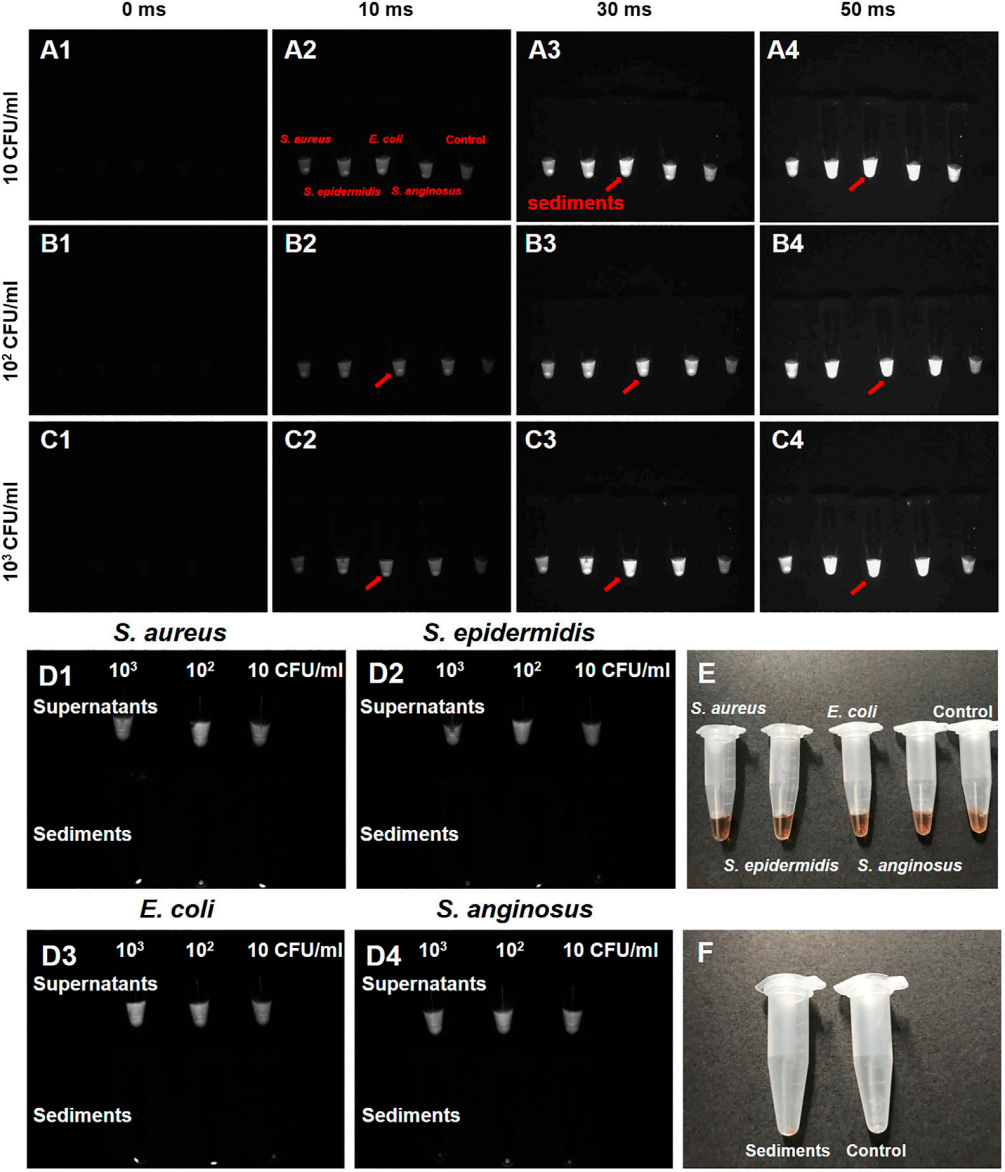
**(A–C)** NIR-II fluorescence images of four bacteria strains (*S. aureus*, *S. epidermidis*, *E. coli* and *S. anginosus*) co-cultured with PbS QDs under an exposure time of 0 ms, 10 ms, 30 ms and 50 ms at a bacterial concentration of 10 CFU/ml, 10^2^ CFU/ml and 10^3^ CFU/ml in comparison with a control group (red arrow: sediments presenting high fluorescence signal). **(D1–D4)** NIR-II fluorescence images of supernatants and bacterial sediments after co-culture. **(E)** Bright-field photograph of **(A)**. **(F)** Bright-field photograph comparing the bacterial sediments and a control group after co-culture.

The labeling efficiency of PbS QDs was then investigated with *S. aureus*, *S. epidermidis*, *E. coli* and *S. anginosus in vitro* (Figure 2). Bacterial suspensions of the four typical bacterial strains were first diluted into different concentrations. Then, a 50 μl portion of each bacterial suspension at three different concentrations (10, 10^2^ and, 10^3^ CFU/ml) was mixed with 50 μl of PbS QDs and co-cultured at 37°C under 5% CO_2_ for 1 h respectively. After centrifugation, NIR-II fluorescence imaging was adopted for these mixtures under an exposure time of 0 ms, 10 ms, 30 ms and 50 ms in comparison with a control group (100 μl of pure PbS QDs). As shown in Figures 2A–C, fluorescence signals detected in each sample increased with exposure time. However, it was noted that a bright spot with high fluorescence signals was demonstrated at the bottom of the tube in samples with bacteria, compared with the control group which was a uniform liquid. Therefore, it was suggested that an aggregation of bacteria labeled with PbS QDs was formed after centrifugation.

To further confirm the results, the original supernatants were separated, leaving the sediments rinsed by PBS till the supernatants became colorless. From the NIR-II images shown in Figure 2D, fluorescence signals could be detected in all supernatants of the four bacteria at the three concentrations, indicating relatively excessive PbS QDs against bacteria. On the other hand, comparatively high fluorescence signals were detected in the bacterial sediments. As pure PbS QDs did not form sediments after centrifugation at preparation, the current sediments were considered to be bacteria labeled with PbS QDs after co-culture. According to previous studies, the labeling capability of our PbS QDs could be explained by electrostatic interaction between the functional groups outside PbS QDs and the surface of the bacterial cell wall (Kloepfer et al., 2003; Hahn et al., 2005; Chen et al., 2015). Therefore, it was proved that our PbS QDs could label bacteria with a high efficiency *in vitro*, further presenting a promising imaging probe for bacteria detection *in vivo*.

### *In Vivo* Real-Time NIR-II Fluorescence Imaging of Bacterial Infection

*In vivo* bacteria imaging is crucial for detecting bacterial infections, not only ensuring an early diagnosis but also facilitating monitoring the development of an infection over time. To test the *in vivo* imaging potential of our prepared PbS QDs, nude mice were subcutaneously infected with 25 μl of bacteria on the back at three sites away from another at a bacterial concentration of 10^12^ CFU/ml, 10^8^ CFU/ml and 10^4^ CFU/ml. Subsequently, the same amount of PBS was injected at a fourth site as a control group. Then, 50 μl of PbS QDs was injected subcutaneously at the same four sites. After that, NIR-II fluorescence images were recorded at 10 min, 1 h, 2 h, 1 day, 2 days, 1 week, 2 weeks and 3 weeks post-infection.

**FIGURE 3:**
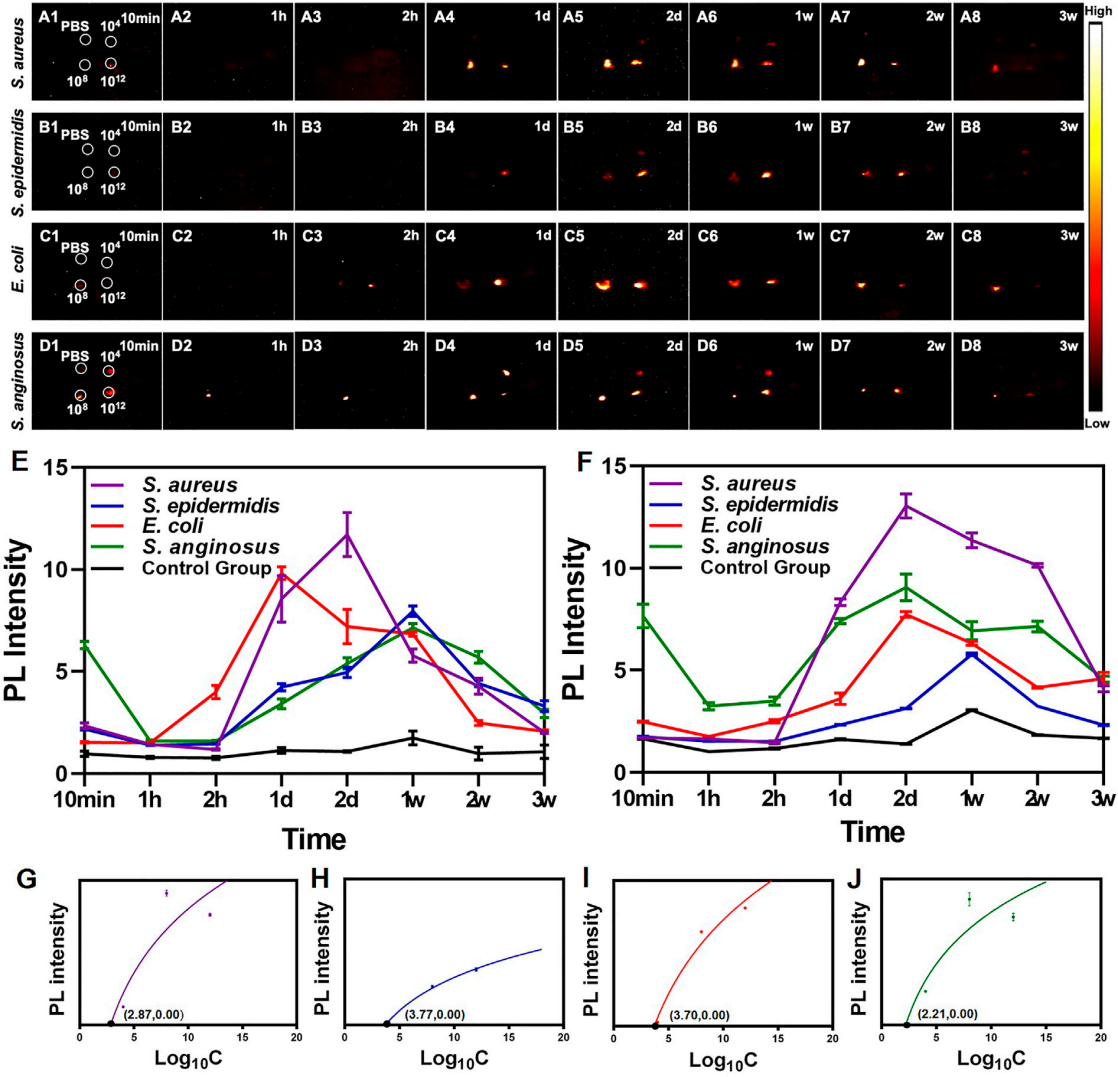
**(A–D)** NIR-II images of nude mice at 10 min, 1 h, 2 h, 1 day, 2 days, 1 weeks, 2 weeks and 3 weeks post-infection. **(E)** PL intensity of the site injected with 10^12^ CFU/ml of bacteria. **(F)** PL intensity of the site injected with 10^8^ CFU/ml of bacteria. Graphs depicting the relevance between PL intensity and different concentration of four strains of bacteria including **(G)** *S. aureus*, **(H)** *S. epidermidis*, **(I)** *S. anginosus* and **(J)** *E. coli*.

As shown in Figures 3A–D, after the injection of PbS QDs, generally higher fluorescence signals were detected at the bacteria-infected sites compared with the control group. It was suggested that PbS QDs rapidly labeled bacteria at the bacteria-infected sites, immediately showing high fluorescence signals. In contrast, the pure PbS QDs were diluted by PBS when mixed subcutaneously, resulting in a decrease in PL intensity with a faster rate of clearance. Furthermore, PL intensity at the site infected with 10^12^ CFU/ml (Figure 3E) and 10^8^ CFU/ml (Figure 3F) of bacteria as well as the control group were measured. A wave pattern of PL intensity was shown at the bacteria-infected sites across the four bacteria strains, while a relatively flat line of PL intensity was displayed by the control group. The differences in the changing pattern of PL intensity further confirmed that the PbS QDs labeled bacteria *in vivo*.

Remarkably, the changing pattern of PL intensity of the PbS QDs labeled bacteria at the infected sites reflected a real-time bacterial load *in vivo*, which in turn demonstrated the process of development of bacterial infection. After the bacteria were injected and labeled with PbS QDs subcutaneously, there was firstly a decrease of PL intensity, then followed with an increase up to a peak, and finally the fluorescence signals became undetectable over time. As is known to all, short after the occurrence of a bacterial infection, the invading bacteria will be first attacked by the innate immune system (Albiger et al., 2007; Nizet, 2007), leading to a reduction of bacterial load. However, as the eradication of most bacteria mainly relies on the adaptive immune response which is stimulated in the later stage of bacterial infection, an interval for clonal expansion of lymphocytes will allow for bacterial propagation, resulting in a resurge of bacterial load (Albiger et al., 2007; Hall et al., 2014). For those with a robust immune system, the bacteria at the infected site will be gradually eliminated and eventually cleared out of the body, resulting in a drop of bacterial load. As the changing pattern of bacterial load was in consistent with the observed PL intensity of PbS QDs, it was suggested that PbS QDs not only successfully detected and labeled bacteria *in vivo*, but also could reflect and monitor the bacterial load *in vivo*.

Furthermore, the relationship between bacterial load and PL intensity *in vivo* were further analyzed. As shown in Figures 3G–J, the four strains of bacteria shared the same shape of logarithmic curves, showing increasing PL intensity with bacterial load under a certain bacterial concentration and gradually became flat. It was reported that high concentration of bacteria would inhibit the electrostatic interaction between the functional groups of PbS QDs and bacterial membrane due to steric effects, which might be due to the mechanism that the smaller interspace could make it difficult to realize further groups interactions in the labeling process (Albiger et al., 2007). Nevertheless, the detection limit of *S. aureus*, *S. epidermidis*, *E. coli* and *S. anginosus in vivo* was calculated as 10^2.87^, 10^3.77^, 10^3.70^ and 10^2.21^ CFU/ml respectively, indicating a relatively low limit achieved *in vivo*. Therefore, with longitudinal observation, *in vivo* monitoring of the development of bacterial infection could be further achieved, thus facilitating diagnosis and treatment under a real-time and long-time imaging guidance.

### Analysis of Pathology and Inflammatory Factors

**FIGURE 4:**
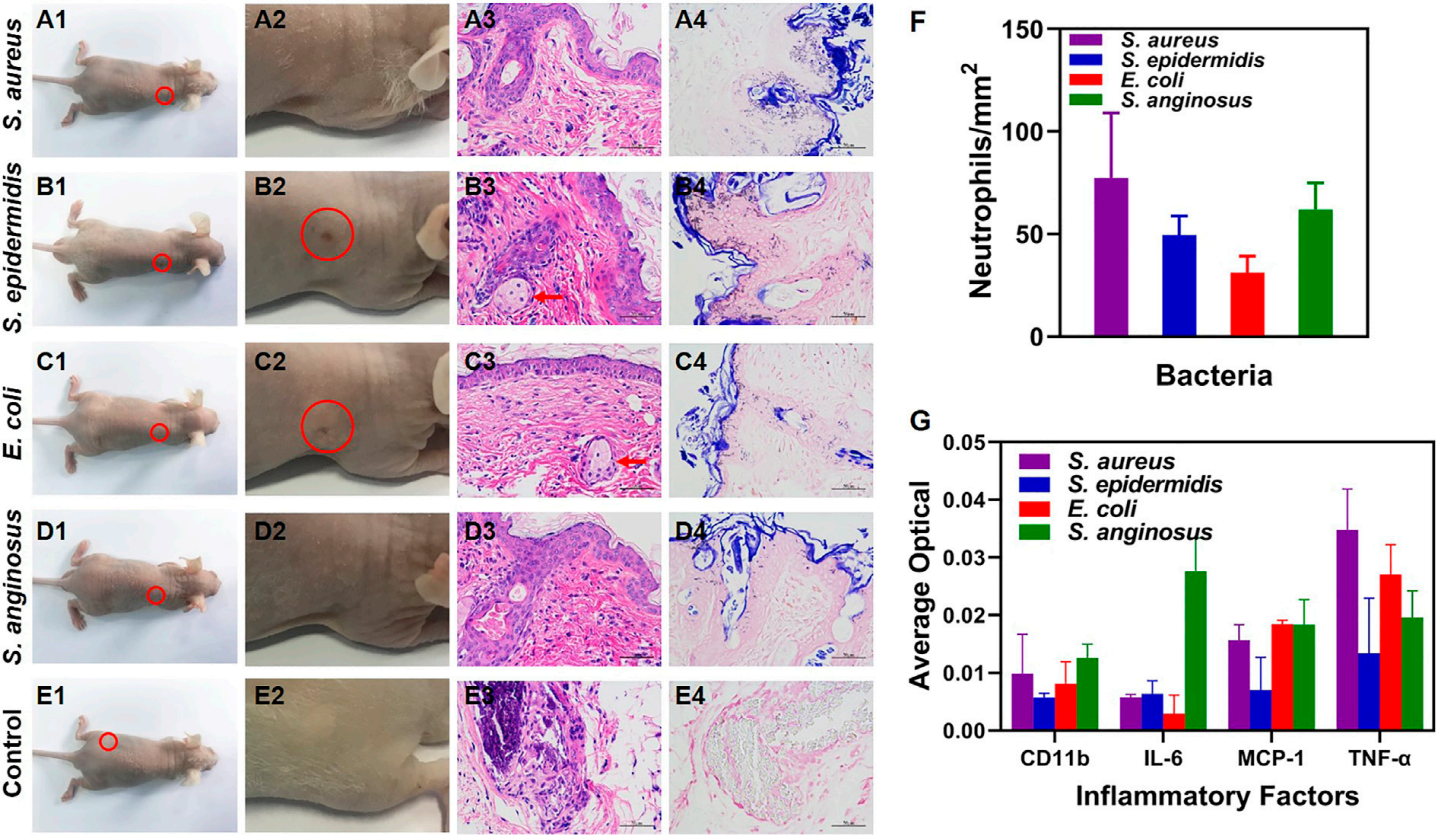
**(A1–E1)** Bright-field photographs of the infected nude mice at 3 w post-infection (**E1** is re-used image of **A1** in which PBS injected site is circled). **(A2–E2)** Corresponding zoomed-in photographs of **(A1–E1)** at the site infected with 10^12^ CFU/ml of bacteria and the control group (red circle: healed skin rupture). **(A3–E3)** H&E staining micrographs of the skin tissues at the site infected with 10^12^ CFU/ml of bacteria (red arrow: foreign body giant cells; × 400 magnification). **(A4–E4)** Gram staining micrographs of the skin tissues at the site infected with 10^12^ CFU/ml of bacteria (×400 magnification). **(F)** Neutrophil count with the H&E staining micrographs. **(G)** Inflammatory factors (CD11b, IL-6, MCP-1 and TNF-α) measured with immunohistochemical micrographs.

At 3 weeks post-infection, the nude mice were photographed right after the NIR-II imaging. An overall view of the whole nude mice was shown in Figures 4A1–E1 and photographs focusing on the site with 10^12^ CFU/ml of bacteria and the control group were shown in Figures 4A2–E2. As shown in Figures 4B2, C2, healed skin lesions were observed at the site infected with *S. epidermidis* (Figure 4B2) and *E. coli* (Figure 4C2), indicating a healing stage of bacterial infection. Furthermore, histological H&E staining was performed to study the histopathology of the infected tissues. The sites infected with 10^12^ CFU/ml of bacteria (Figures 4B3–D3) showed aggregation of neutrophils and appearance of foreign body giant cells, both of which were commonly seen in the late stage of infection. In comparison, the control group showed insignificant inflammatory infiltration (Figure 4E3). Additionally, Gram staining was carried out for bacteria analysis, with Gram-positive bacteria (*S. aureus*, *S. epidermidis* and *S. anginosus*) stained purple and Gram-negative bacteria (*E. coli*) stained pink (Figures 4A4–D4). No bacteria were found in the control group (Figure 4E4). Moreover, neutrophil count (Figure 4F) and analysis of several major inflammatory factors (CD11b, IL-6, MCP-1, TNF-α) (Figure 4G) were done with immunohistochemical slides to assess inflammatory response. There was no significant difference of neutrophil count among the four bacteria strains. As for inflammatory factors, the relatively high expression of TNF-α (mainly secreted by mononuclear macrophages) indicated the involvement of the innate immune system, while the others were at an insignificant level.

### Biosafety Validation of PbS QDs

**FIGURE 5:**
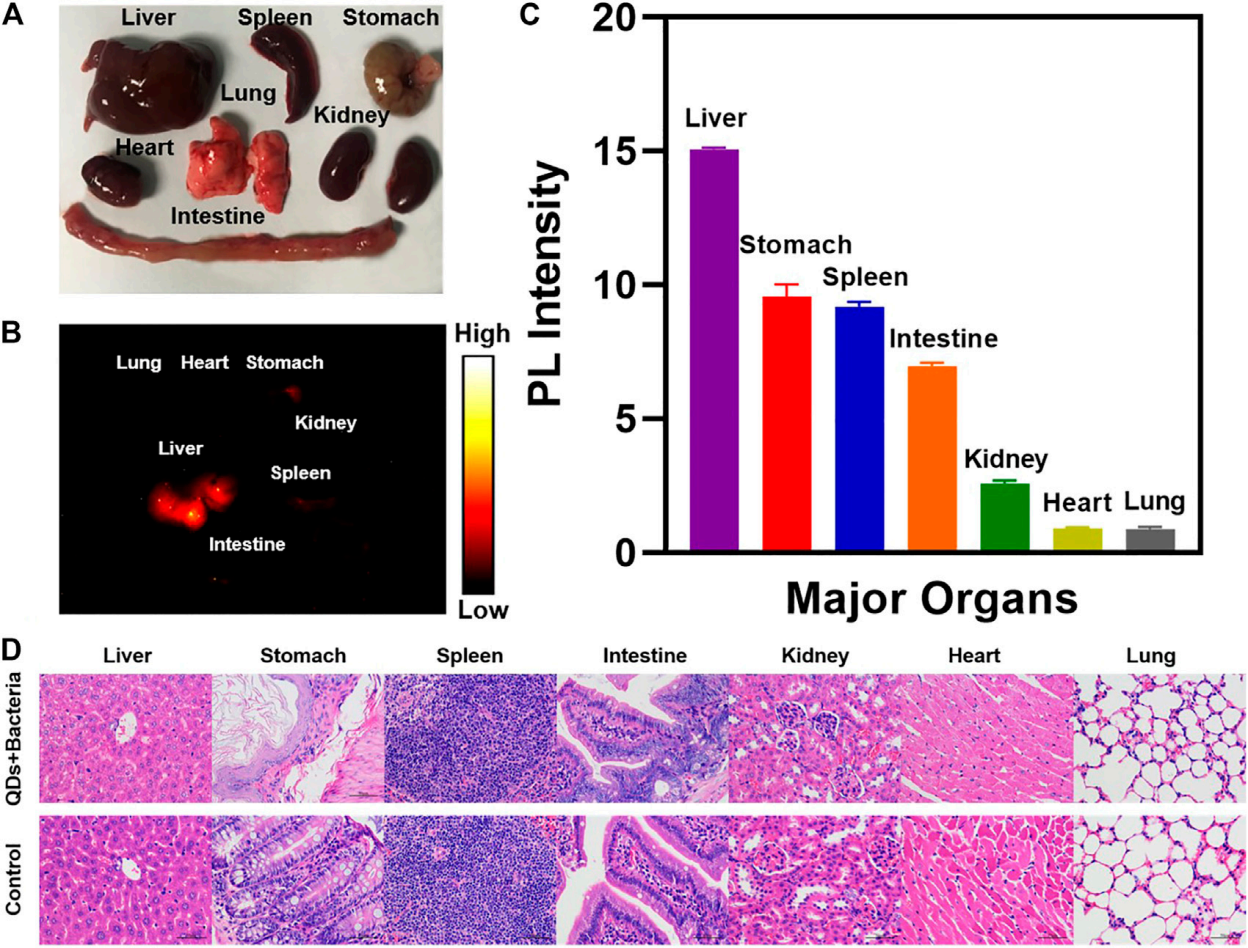
**(A)** Photograph of major organs harvested from the infected nude mouse. **(B)** NIR-II image of major organs harvested at 3 weeks post-infection. **(C)** PL intensity measured from the NIR-II image of the harvested major organs. **(D)** H&E staining micrographs of representative major organ tissues of the infected nude mice compared with a control group (magnification: ×400).

The distribution of PbS QDs in the major organs of the infected nude mice at 3 w post-infection was analyzed in Figure 5. There was no obvious abnormality in major organs from the bright-field photograph (Figure 5A). Among all the harvested organs, fluorescence signals were detected in the liver, spleen, stomach and parts of intestine from the NIR-II image (Figure 5B) and the PL intensity was measured in Figure 5C. It was in agreement with a previous study that major accumulation of PbS QDs was observed in the liver and the spleen after 24 h through intravenous injection (Chen et al., 2016a). As PbS QDs were injected subcutaneously in the current study, the metabolization rate was comparably slow, resulting in longer time for PbS QDs to accumulate in the organs and eventually to be cleared out of the body. From another perspective, the fluorescence signals detected in major organs indicated that the PbS QDs were metabolized by the body and then joined the circulation instead of simply quenched subcutaneously over time. Furthermore, H&E staining micrographs of major organ tissues at 3 w post-infection compared with a control group were shown in Figure 5D. No obvious signs of tissue injury or inflammation were noted.

## Conclusion

In conclusion, a novel imaging strategy based on highly fluorescence PbS QDs with NIR-II emission was developed for real-time *in vivo* detection of bacterial infection. After a high bacteria-labeling efficiency was proved *in vitro*, the PbS QDs enabled a rapid detection and imaging of bacteria *in vivo*, with a detection limit around 10^3^–10^4^ CFU/ml, allowing a real-time investigation on the bacterial load. Besides, the biocompatible PbS QDs were eventually metabolized and cleared out of the body with no noticeable abnormalities in major organs. Our work demonstrated a highly efficient imaging strategy for detection and monitoring of bacterial infection *in vivo*, facilitating the optimization of anti-infection treatment according to dynamically changing bacterial load under a real-time imaging guidance.

## Data Availability

The raw data supporting the conclusions of this article will be made available by the authors, without undue reservation.
